# Morphological and Genetic Diversity of Rhizobia Nodulating Cowpea (*Vigna unguiculata* L.) from Agricultural Soils of Lower Eastern Kenya

**DOI:** 10.1155/2017/8684921

**Published:** 2017-12-31

**Authors:** Damaris K. Ondieki, Evans N. Nyaboga, John M. Wagacha, Francis B. Mwaura

**Affiliations:** ^1^School of Biological Sciences, University of Nairobi, P.O. Box 30197-00100, Nairobi, Kenya; ^2^Department of Biochemistry, University of Nairobi, P.O. Box 30197-00100, Nairobi, Kenya

## Abstract

Limited nitrogen (N) content in the soil is a major challenge to sustainable and high crop production in many developing countries. The nitrogen fixing symbiosis of legumes with rhizobia plays an important role in supplying sufficient N for legumes and subsequent nonleguminous crops. To identify rhizobia strains which are suitable for bioinoculant production, characterization of rhizobia is a prerequisite. The objective of this study was to assess the morphological and genetic diversity of rhizobia that nodulates cowpea in agricultural soils of lower eastern Kenya. Twenty-eight rhizobia isolates were recovered from soil samples collected from farmers' fields in Machakos, Makueni, and Kitui counties in lower eastern Kenya and characterized based on morphological characteristics. Thirteen representative isolates were selected and characterized using BOX repetitive element PCR fingerprinting. Based on the dendrogram generated from morphological characteristics, the test isolates were distributed into two major clusters at a similarity of 75%. Phylogenetic tree, based on BOX repetitive element PCR, grouped the isolates into two clusters at 90% similarity level. The clustering of the isolates did not show a relationship to the origin of soil samples, although the isolates were genetically diverse. This study is a prerequisite to the selection of suitable cowpea rhizobia to develop bioinoculants for sustainable crop production in Kenya.

## 1. Introduction

Rhizobia are soil bacteria that infect roots of leguminous plants to form nodules, where they differentiate and fix atmospheric nitrogen (N) for the advantage of the plant [1]. Nitrogen is an important nutrient required for growth and development of plants. Its deficiency in the soil as well as in the crop adversely affects plant growth and yield in smallholder farms in Africa [2]. Legumes rely on biological fixation of nitrogen through symbiotic association with rhizobia to attain the required quantity of N for high grain yields [3]. The legume-rhizobium symbiosis contributes at least 50% of the 175 million tons of N per year in agricultural production [4]. This process is a sustainable and cost-effective strategy of adding N to terrestrial ecosystem in African smallholder farming systems.

Cowpea (*Vigna unguiculata* L.) is an annual legume crop widely grown in East Africa [5]. It is the third most important legume crop grown in Kenya, after beans and pigeon peas [6]. The crop has gained importance as a source of dietary protein to small-scale farmers in Kenya. Due to its ability to fix nitrogen into the soil, it is grown in mixed intercropping systems with no application of chemical fertilizers [7]. However, information on diversity of cowpea-nodulating rhizobia in lower eastern Kenya soils is limited, although it has huge potential in management of soil fertility that results in increased crop production. Given the variation in physicochemical properties of the soils of lower eastern Kenya [8], selection of efficient strains of rhizobia for cowpea nodulation in different soils is an important step towards yield improvement. Thus, evaluation of morphological and genetic diversity of native strains of rhizobia represents an important prerequisite to obtaining novel inoculants.

Given that rhizobia are taxonomically diverse [9], efficient methods of classifying isolates are necessary to identify strains with high nitrogen-fixation ability [10]. Initial characterization and screening of rhizobia have been based on morphological features; however, this method is highly prone to errors due to morphological plasticity. Information on diversity of rhizobia can be improved by combining both morphological analyses and genotypic differences between strains. Several molecular techniques including random amplified polymorphic DNA [11], restriction fragment length polymorphism [11, 12], and repetitive extragenic palindromic-polymerase chain reaction (rep-PCR and BOX-PCR) [13–15] have been applied for identification and genetic diversity studies of rhizobia. The repetitive extragenic palindromic PCR procedure has been successfully used to characterize bacteria; it has proved to be faster, easy, and reproducible with high potential of differentiating isolates at the level of strains [16, 17]. In addition, this technique yields good results for correlation with pairwise DNA-DNA analyses [18].

To date, no study has been done on the range of morphological and genetic diversity of indigenous rhizobia from agricultural soils of lower eastern Kenya. The identification of native rhizobia strains well adapted to local environmental conditions and edaphic characteristics to be used as bioinoculants of cowpea in Kenyan soils could have a significant economic and environmental impact. Considering the economic importance of cowpea and the lack of studies on rhizobia populations of cowpea in soils of lower eastern Kenya, bacteria from cowpea nodules grown in soils of lower eastern Kenya were isolated and characterized. The objectives of this study were to (i) assess the morphological characteristics of cowpea-nodulating rhizobia isolated from soil samples from farmers' fields in lower eastern Kenya and (ii) evaluate the genetic variability of cowpea rhizobia isolates using BOX-PCR fingerprinting.

## 2. Materials and Methods

### 2.1. Description of Field Sites and Soil Sampling Procedures

Eighteen soil samples were collected from selected agricultural fields, which have never been inoculated with rhizobia, in Machakos, Kitui, and Makueni counties of lower eastern Kenya. The three counties are characterized by semiarid lands with highly weathered soils that have low organic matter and are low in productivity. High temperatures and low annual rainfall dominate the sampling areas (Table 1). At least five fields were randomly selected in every county. Using a hand shovel, soil (1 kg) was collected from each field to a depth of 5 cm from each sampling point. The hand shovel was cleaned after each sampling with running tap water and dried using sterile cloth. The soil samples were mixed thoroughly to form a composite sample. The soil samples were placed in plastic polythene bags, labeled, and stored at room temperature (23 ± 2°C) for subsequent isolation of rhizobia.

### 2.2. Isolation of Rhizobia Associated with* Vigna unguiculata*

The soil samples were placed in labeled plastic pots (5 cm diameter, 250 g) and certified cowpea* (Vigna unguiculata)* cultivar KV 271 was used as a trap plant in the glasshouse. Cowpea variety KV 271 is a local landrace with seeds that are maroon in color, preferred by farmers in lower eastern Kenya due to its high yield, disease resistance, and superior cooking quality and taste. The collected soil samples were placed in labeled plastic pots and watered until adequately wet with tap water. Four cowpea seeds were sown in triplicate for each soil sample and thinned to two seedlings per pot 7 days after planting. The remaining two plants served as rhizobia trap plants. The pots were placed on a table in the glasshouse and watered whenever needed. The experiment had a completely random design. The plants were uprooted 60–70 days after planting, roots were washed using tap water, and the nodules were detached. The collected nodules were immediately allowed to dehydrate in glass bottles with silica gel, a thin layer of cotton, and screw cap-sealed [19].

Rhizobia isolation from cowpea nodules was done following the method described by Somasegaran and Hoben [20]. Healthy, unbroken, and pink root nodules from each of the soil samples were randomly selected for isolation of rhizobia. Nodules were surface sterilized in a laminar flow cabinet by immersion in ethanol (95% v/v) for 30 seconds followed by 4-minute immersion in 3.8% sodium hypochlorite and finally rinsed with six changes of sterile double-distilled water. The nodules were crushed in 100 *μ*l of sterile distilled water using a sterilized blunt forceps. One loopful of each nodule suspension was aseptically streaked onto YEMA (10 g mannitol, 0.2 g MgSO_4_·7H_2_O, 0.2 g NaCl, 0.5 g K_2_HPO_4_, 1 g yeast extract, and 15 g agar, pH 6.8 ± 0.2) medium containing bromothymol blue. The plates were wrapped with aluminium foil (for darkness), incubated at 28°C, and observed daily for the period necessary to characterize colony growth [21].

### 2.3. Morphological Characterization of Rhizobia Isolates

Morphological characterization of the rhizobia was done to determine their growth rate (slow or rapid), mucous production (quantity of mucous and elasticity), change in pH of the medium during growth of the isolates, and colony characteristics. The formation of colonies on YEMA plates was monitored daily for 10 days, and the pH change of the growth medium was scored on YEMA plates containing 0.25 mg/l bromothymol blue (BTB). The cultures were incubated for 10 days at 28°C and observed for color change on a daily basis. The isolates that turned the growth medium to yellow were acid producers and classified as fast growers. The isolates that turned the medium to blue were considered alkaline producers and were classified as slow growers. After incubation at 28°C for 2 to 10 days, distinct colonies were characterized based on their size (small: <2 mm, medium: 2-3 mm, large: 4-5 mm), color (white/milky and transparent), shape (round, ellipsoid), transparency, borders, and elevation (convex, raised, flattened, umbonate).

### 2.4. Gram Staining and Ability to Absorb Congo Red

Gram staining and microscopy were carried out to determine if the cultures were Gram negative or positive. Staining was done following the method described by Harold [22]. A colony of bacterial culture was picked with a sterile inoculating wire loop, and a thin smear was prepared in a drop of water on a clean glass slide. The smear was air-dried, heat fixed, stained with crystal violet for one minute, and then washed with distilled water. The smear was flooded with iodine solution for one minute followed by one-minute decolorization with ethanol (95% v/v), then washed with distilled water to stop the action of alcohol, and counterstained with safranin for 20 minutes. The slide was washed with distilled water, dried, and observed under light microscope at 1000x magnification using oil immersion. The ability of the isolates to absorb Congo red was tested by adding 1% Congo red solution on prepared and autoclaved YEMA media before pouring into sterile Petri plates.

### 2.5. Genetic Variability of Rhizobia Isolates

Thirteen out of the 28 rhizobia isolates recovered were selected for further molecular variability studies based on cluster analysis of morphological characteristics.

### 2.6. DNA Extraction from Rhizobia Suspension Cultures

Genomic DNA was isolated from rhizobia cultures using cetyltrimethylammonium bromide (CTAB) method described by Wilson [23]. The isolated DNA was dissolved in sterile water. The quality and integrity of DNA were confirmed by agarose electrophoresis in 1x Tris-acetate-EDTA (TAE) and quantification was done using a spectrophotometer (Beck Man Coulter UV/VIS, USA). The bands were visualized and photographed using DNR-Imaging System. The DNA was stored at –20°C till use.

### 2.7. Repetitive Element Sequence-Based PCR Analysis

Thirteen rhizobia isolates were analyzed using BOX-PCR fingerprinting. Amplifications were performed using BOXA1R primer (5′-CTACGGCAAGGCGACGCTGACG-3′) described by Versalovic et al. [14]. A total volume of 20 *μ*l was used for amplification reactions. The PCR reaction contained 100 ng of genomic DNA, 1 *μ*l BOXA1R primer, 4 *μ*l PCR premix (dNTPs, PCR buffer, Taq polymerase), and final volume adjusted to 20 *μ*l using sterile double-distilled water. The following thermocycler conditions were used: initial denaturing at 94°C for 5 minutes, 30 cycles of denaturing at 94°C for 1 minute, annealing at 52°C for 1 minute, extension at 65°C for 8 minutes, and final extension at 65°C for 15 minutes before cooling at 4°C. The amplification products were analyzed using 2% (w/v) agarose gel electrophoresis at 80 Volts for 150 minutes. The gel was visualized and photographed using the DNR-Imaging System.

The consistency of the banding patterns was verified by repeating the PCR reactions at least three times for each of the rhizobia isolates. Only distinct and reproducible bands for each sample were scored and used to generate binary matrix in which the presence of a band was scored as one (1) and absence of a band as 0 (zero).

### 2.8. Data Analysis

Morphological and cultural characteristics of rhizobial colonies were scored numerically and the data obtained was subjected to a hierarchical cluster analysis using the squared Euclidean distance similarity and between-groups linkage procedures using SPSS software version 20.

The amplified products were scored for presence (1) and absence (0) of a band. Molecular diversity analysis was done using unweighted pair group mean arithmetic (UPGMA) algorithm method and Jaccard coefficient to generate/construct a dendrogram.

## 3. Results

### 3.1. Isolation and Identification of Rhizobia Isolates

**Figure 1:**
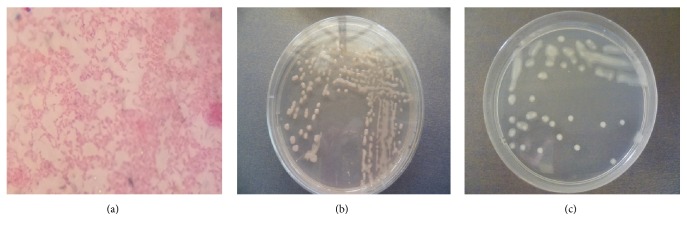
Gram-negative reaction and growth response of rhizobia isolates on YEMA medium containing Congo red. Gram-negative reaction of a fourteen-day-old rhizobia isolate (a); colonies of rhizobia isolate on YEMA media with Congo red (b) and without Congo red (c).

Twenty-eight isolates of rhizobia were recovered from root nodules of* V. unguiculata* grown on agricultural soils collected from three counties of lower eastern Kenya (Table 1). All the 28 rhizobia isolates were Gram-negative and rod-shaped cells (Figure 1). All isolates absorbed little Congo red to produce colonies that were pale pink to whitish in color.

### 3.2. Morphological Characteristics of Rhizobia Isolates

**Figure 2:**
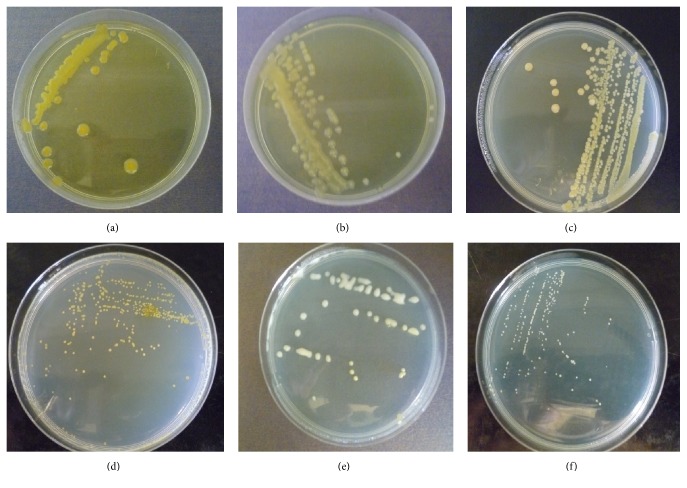
Colonies of rhizobia isolates on yeast mannitol agar medium containing bromothymol blue. (a) Yellow with creamy and smooth margins, medium sized, round, (b) cream with smooth margin, medium sized, round, (c) yellowish, smooth margins, medium sized, round (d) yellow, smooth margins, small sized, punctiform, (e) white, smooth margins, medium sized, round (f) white, smooth margins, small sized, punctiform.

When incubated at 28°C, 55% of the isolates were fast-growing, while 45% were slow-growing. On YEMA medium containing bromothymol blue (BTB), fast-growing strains of rhizobia produced colonies that were either yellow with creamy margins or yellowish or cream ranging from 2–5 mm in diameter within 3 days of incubation (Figure 2). Slow-growing isolates produced white colonies, but in some cases the colonies were milky and translucent or yellow with diameter range <2-3 mm after incubation for 7–10 days. The shapes of the colonies in most isolates were round except a few isolates which had punctiform and some were irregular. The margins were smooth in many isolates or undulated in a few isolates. The pH change of culture medium as indicated by BTB was acidic for fast-growing isolates except isolate MC4 from soil collected in Machakos county which alkalized the medium. Slow-growing isolates alkalized the medium except isolate KT2 from Kitui county that acidified the medium.

Regarding mucus production, 20 isolates (MC1, MC5, MC6, MC14, MC7, MC13, MC12, MK11, MK9, MK12, MK10, KT5, MC2, KT4, MC8, MC10, MC9, MC4, MC11, KT6) had high mucus production while 8 isolates (KT3, KT2, MK20, MK18, MK15, MC3, KT1, MC15) had intermediate mucus production. There were no dry colonies. The appearance of the isolates varied with most of the isolates being diffuse and nonelastic (KT5, MC14, MC7, MC13, MC12, MK12, MK11, KT5, MK9, MK20, KT4, KT2, MC10, MC9, MC4, MK10, MC8, MC11, MC1, MC5, MK20, MC6, MC3, MC2) while others were dense and elastic in appearance (KT3, MC15, KT1, MK18).

### 3.3. Analysis of Morphological Characteristics of the Rhizobia Isolates

**Figure 3:**
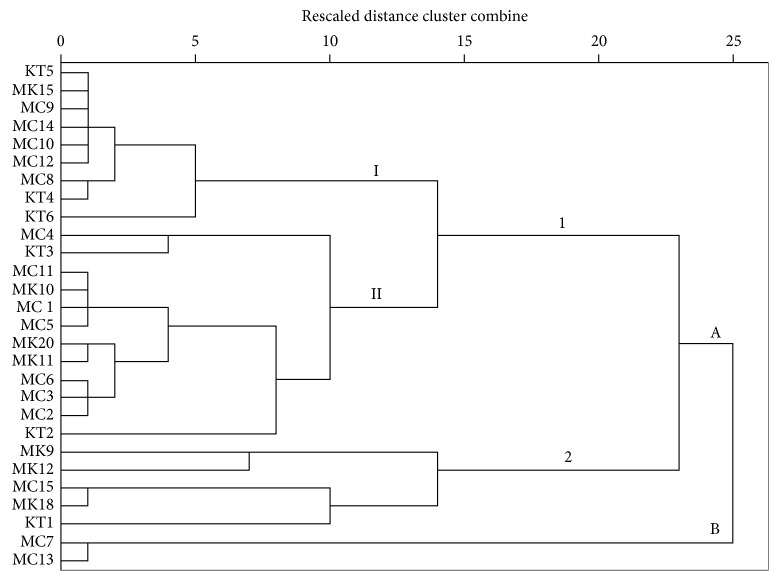
Dendrogram showing the phenotypic relationships generated among rhizobia isolates from agricultural soils of lower eastern Kenya. The UPGMA method was used for the cluster analysis of seven phenotypic characteristics (color, form, size, margin, elevation, growth rate, and pH).

The phenotypic characteristics of the rhizobia isolates were compared by cluster analysis. The resulting dendrogram separated the rhizobia isolates into two main clusters, A and B at a similarity level of 75% (Figure 3). Cluster B consisted of only two isolates (MC7 and MC13) that were slow-growing, milky, irregular, and translucent. Cluster A consisted of many isolates with two subclusters, 1 and 2. Subcluster 1 consisted of two further large groups, I and II. Cluster A subcluster 1 group I consisted of slow-growing isolates (KT5, MK15, MC9, MC14, MC12, MC10, MC8, KT4, KT6) that were white in color and medium sized with smooth margins and alkalized YMA medium containing BTB. The average similarity for the isolates ranged from 95% (for isolate KT6) to 98% (for isolates KT5, MK15, MC9, MC14, MC10, MC12, MC8, KT4).

Cluster A subcluster 1 group II consisted of fast-growing isolates except KT2 isolate that was slow-growing but acidified the medium and was yellow in color. The average similarities for the isolates ranged from 90% to 98%. Subcluster 2 consisted of fast-growing isolates (MK9, MK12, MC15, MK18, KT1) that had mixed characteristics with some being yellow with creamy margins (MK9, MK12, KT1) or yellow (MK18 and M15), irregular (MK12 and MK9) or round (MK18, MC15, KT1) in shape, and large in size (MK9, MK12, KT1).

### 3.4. Genetic Variability of Rhizobia Isolates by BOX-PCR

**Figure 4:**
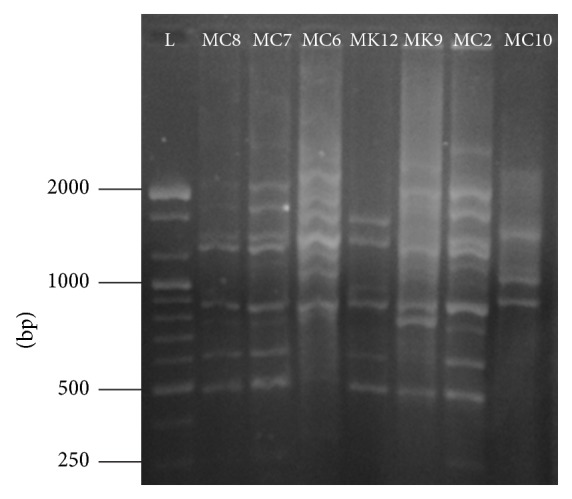
Agarose gel electrophoresis of DNA fingerprint patterns of rhizobia isolates generated in the reactions with BOXA1R primer.

**Figure 5:**
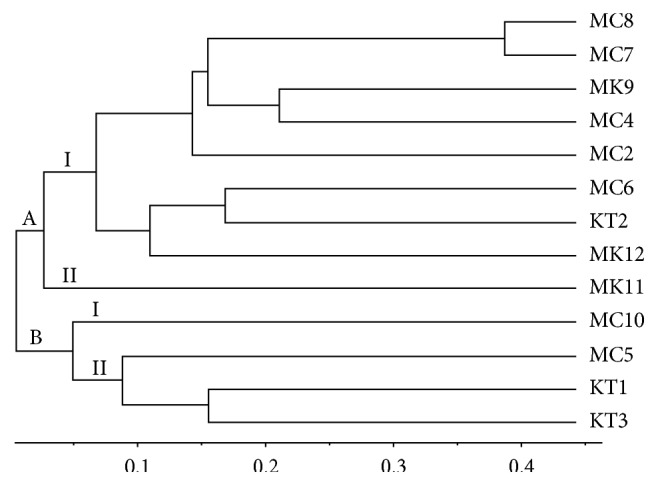
Dendrogram showing the genetic relationship of rhizobia isolated from soils of lower eastern Kenya after analysis of binary matrix data of BOX-PCR products using UPGMA method and Jaccard's similarity coefficient.

Characteristic fingerprint patterns were produced by all rhizobia isolates with distinct and scorable bands ranging from 250 to 2500 bp and their numbers varied from 4 to 12 bands per isolate (Figure 4). PCR was repeated at least three times using different DNA samples from the same isolate and only those with reproducible bands and similar fingerprints were used for analysis. Dendrogram of similarity generated from data obtained from BOX-PCRs on the investigated isolates separated the rhizobia isolates into two main clusters, cluster A and cluster B (Figure 5). Cluster A consisted of nine isolates (MC8, MC7, MK9, MC4, MC2, MC6, KT2, MK12, and MK11) obtained from soils collected from all the three counties, while cluster B contained four isolates (MC10, MC5, KT1, and KT3) from Machakos and Kitui counties. The genetic similarity level between the clusters was 100%.

Cluster A is divided into two subclusters I and II with a genetic similarity of 98%. Subcluster I contained 8 isolates from soil samples from the three counties, while subcluster II had only one isolate (MK11) from soil sampled from Makueni county. Cluster B was also divided into two subclusters, subcluster I and II, with a genetic similarity of 95%. Subcluster I contained only one isolate (MC10) from Machakos county. Subcluster II contained three isolates (MC5, KT1, and KT3) from soil sampled from Machakos and Kitui counties and one isolate (MC10) from Machakos county.

## 4. Discussion

This study reports on the morphological and genetic variability of bacteria nodulating cowpea in soil samples collected from Machakos, Makueni, and Kitui counties of lower eastern Kenya, which is characterized by high thermal amplitudes and low rainfall. Twenty-eight isolates of rhizobia were recovered from root nodules of cowpea plants. These isolates were designated as rhizobia on the basis of their colony characteristics, cell morphology, and inability to absorb Congo red dye. All the isolates were Gram-negative and had rod-shaped cells. In addition, all the isolates cultured on YEMA medium containing Congo red dye produced colonies that were whitish to pale pink indicating that the isolates did not absorb the dye when incubated in the dark. The inability of the isolates to absorb Congo red dye is a distinctive character of rhizobia [24]. Species of rhizobia do not absorb Congo red dye or may absorb little amount to give a pale pink appearance [20, 24]. Shoukry et al. [25] also observed whitish or pale pink colonies of faba bean rhizobia isolates on YEMA media containing Congo red. However, there are exceptions of rhizobia strains that can absorb Congo red depending on age of culture, concentration of the dye, and exposure to light [20] to produce orange or deep pink colonies, for example, species of* Burkholderia *[26].

Based on the growth rate on YEMA medium, the* Rhizobiaceae* family of bacteria can be divided into two major groups, namely, fast- and slow-growing rhizobia. In the present study, both groups of rhizobia were observed in all the soils from the three counties. This finding concurs with earlier studies [27–29] that reported the appearance of both fast- and slow-slowing rhizobia in many subtropical and tropical soils. The results also showed that cowpea was nodulated by both groups of rhizobia, but fast growers formed the majority. Fifty-five percent of rhizobia isolates in this study formed colonies on YEMA media within three days of incubation and were therefore classified as fast growers according to Odee et al. [30]. The colonies formed by these fast-growing isolates in the present study were yellow with creamy margins, convex, round with entire margins of size ranging from 2 to 5 mm. Isolates of rhizobia forming colonies within 72 hours have been reported by Singh et al. [31] on soybean and Ngakou et al. [32] on Bambara groundnut, cowpea, and soybean. Slow-growing isolates formed colonies after 7–10 days of incubation that were small to medium sized, white or milky, and translucent and were raised with smooth margins. These are characteristics of* Bradyrhizobium *spp. as described by Howieson and Dilworth [26]. Sanginga et al. [27] reported that slow-growing strains of rhizobia dominate in tropical soils; however, results from the current study showed the reverse with 55% of isolates being fast-growing species. The isolates in this study had yellow and white colonies with milky appearance, while a few isolates showed transparent colonies on YEMA medium containing bromothymol blue. According to Jordan [33], colonies of rhizobia are white, yellow, or pink in color, although it is rare to find yellow or pink colonies of rhizobia [34].

Based on the growth of rhizobia on YEMA medium supplemented with BTB (pH indicator), the isolates were classified into two groups, namely, acid producers and alkalizers. The results indicated that both groups of rhizobia nodulated cowpea and the acid producers were more prevalent than alkalizers. Fifty-five percent of the isolates turned the color of YEMA media supplemented with BTB to yellow within five days of incubation indicating that they are acid producers and hence confirmed to be fast growers as described by Jida and Assefa [35] with the exception of MC4 that alkalized the media. Fast-growing strains of rhizobia confirmed to acidify YEMA medium supplemented with BTB have been isolated from other legumes including Bambara groundnuts, peanut, and cowpea [36, 37]. This frequency of fast-growing isolates was too high for cowpea plant commonly considered to be nodulated by bacterial species of* Bradyrhizobium *that consist of slow-growing strains with the ability to alkalize the media [38]. These findings probably indicate that cowpea nodulation dose not only involve the species of* Bradyrhizobium *but also other species of rhizobia. The findings in this study concur with those reported by Zhang et al. [11] who isolated fast-growing species of rhizobia from cowpea plants. In addition, the high number of fast-growing isolates in this study could be due to the fact that rapidly growing rhizobia are more common in arid and semiarid lands, characteristic of the soil sampling sites in the three counties of lower eastern Kenya. Rhizobia from these regions have the ability to multiply fast within short rains and are more tolerant to stress conditions than slow-growing strains [39], and these survival strategies could explain their greater frequency in soil samples used in this study.

All the slow-growing isolates raised the pH of the medium except isolate KT2 that acidified the medium which is an unusual behavior of slow-growing isolates. This observation indicates that cowpea can form symbiotic associations with bacteria harbouring different culture characteristics. These results concur with those of Zilli et al. [19] who reported slow-growing strains of rhizobia that were acid producers. Both fast- and slow-growing isolates showed mucus production that ranged from high to intermediate with some isolates being dense and elastic and others diffuse and nonelastic. Mucus production probably represents a mechanism of rhizobia adaptation and endurance in hostile climatic and edaphic conditions. It prevents desiccation of the bacteria and helps them withstand fluctuations in temperature, salinity, and acidity [40]. Batista et al. [41] noted increased production of mucus in isolates of* Bradyrhizobium *as a mechanism of adaptation to acid soils of Cerrado region in Brazil. Mucus production by most rhizobia isolates is a fundamental characteristic that is associated with nodulation [41]. This suggests that rhizobia isolates with high mucus production ability have high competitive advantage in the initial infection, colonization, and root nodules formation.

Genomic fingerprinting based on BOX-PCR was used to understand the genetic diversity of rhizobia since it is fast, easy, and reproducible with high discrimination at the level of strains [42]. Amplification of sequence-related BOX elements provides fingerprints to establish phylogenetic relationship among the different strains of bacteria [43]. In this study, the BOX-PCR analysis showed high polymorphism among the tested isolates. Most of the isolates produced unique banding pattern indicating high variability among the isolates. This clearly shows the high genetic variability among rhizobia isolates from agricultural soils of lower eastern Kenya. These findings agree with reports from other parts of the world, in which high molecular diversity of rhizobia was observed in cultivated lands [44]. High genetic diversity in cultivated soils can be due to high demand for nitrogen by the plants, which in turn stimulate nodulation resulting in rhizobia proliferation [45]. Tian et al. [46] also reported high molecular diversity of rhizobia isolated from* Vicia faba* using BOX-PCR. The high rhizobia diversity observed in soils from the three counties might be due to earlier cultivation of legumes belonging to similar cross-inoculation groups as cowpea.

Use of UPGMA algorithm and Jaccard's similarity coefficient for cluster analysis showed profiles that were joined at low similarity level of 10%. These results concur with previous reports by Torres et al. [47], Menna et al. [48], and Binde et al. [49] who observed high variability among rhizobia isolates using BOX-PCR with low similarity level of 10–20% in cluster analysis. The genetic characterization of rhizobia isolates using BOX-PCR procedure was therefore effective for differentiating the isolates used in this study. The presence of a high diversity of isolates in any soil increases the probability for several legume crops to find compatible rhizobia for formation of nodules.

Comparing the two dendrograms constructed based on morphological characteristics and genetic profiles, isolates KT3 and MC4, MC7, and MC8 showed differences in their cluster analysis. Isolates KT3 and MC4 were morphologically similar but genetically different, while MC7 and MC8 were morphologically different but genetically similar. Similar findings were reported by De Lyra et al. [50] of rhizobia strains isolated from peanut that had high differences in their cluster analysis based on cultural characteristics and genetic properties based on BOXA1R profiles. These similarity differences can be explained by the fact that morphological variations are more related to abiotic factors than to genetic factors.

## 5. Conclusion

In soils of lower eastern Kenya, the predominant rhizobia isolates were the fast-growing, which acidified the YMA medium and produced high to intermediate exopolysaccharides (mucus production). These are attributes related to survival strategies in semiarid regions. The current findings show the diversity of fast-growing and slow-growing rhizobia isolates that nodulate* V. unguiculata* and suggest morphological variations among* V. unguiculata* strains as selection tools of candidate isolates to be used as biofertilizer. High genetic diversity was observed by BOX-PCR genomic fingerprinting analysis with the isolates showing distinct patterns of fragments ranging from 4 to 12. The genetic characterization of rhizobia using BOX-PCR proved to be an effective method for discriminating the isolates. This study offers important information for further studies on the diversity of and complex interactions among the root-nodule bacteria, the host plant, and environmental factors. The rhizobia isolates characterized in this study need to be screened for their ability to fix nitrogen using different cowpea genotypes and other legumes of economic importance in lower eastern Kenya.

## Figures and Tables

**Table 1:** Characteristics of counties in lower eastern Kenya and identities of rhizobia isolates per county.

County	Altitude (meters)	Annual temperature ranges (°C)	Annual rainfall (mm)	Soil type	No. of isolates	Isolate identities
Machakos	1000–1600	18–26	500–1300	Clay loam	15	MC1–15
Kitui	1100–1700	14–34.7	300–1050	Sand	6	KT1–5
Makueni	1000–2100	12–28	500–1300	Sandy loam	7	MK9–12, MK15, MK18, MK20

*Source*. Kenya Meteorological Department (http://www.meteo.go.ke).
